# A Correspondence between Individual Differences in the Brain's Intrinsic Functional Architecture and the Content and Form of Self-Generated Thoughts

**DOI:** 10.1371/journal.pone.0097176

**Published:** 2014-05-13

**Authors:** Krzysztof J. Gorgolewski, Dan Lurie, Sebastian Urchs, Judy A. Kipping, R. Cameron Craddock, Michael P. Milham, Daniel S. Margulies, Jonathan Smallwood

**Affiliations:** 1 Max Planck Research Group: Neuroanatomy and Connectivity, Max Planck Institute for Human Cognitive and Brain Sciences, Leipzig, Germany; 2 Department of Neurology, Max Planck Institute for Human Cognitive and Brain Sciences, Leipzig, Germany; 3 Child Mind Institute, New York, New York, United States of America; 4 Nathan Kline Institute for Psychiatric Research, Orangeburg, New York, United States of America; 5 Department of Psychology, University of York, Hesslington, United Kingdom; Lyon Neuroscience Research Center, France

## Abstract

Although neural activity often reflects the processing of external inputs, intrinsic fluctuations in activity have been observed throughout the brain. These may relate to patterns of self-generated thought that can occur while not performing goal-driven tasks. To understand the relationship between self-generated mental activity and intrinsic neural fluctuations, we developed the New York Cognition Questionnaire (NYC-Q) to assess the content and form of an individual's experiences during the acquisition of resting-state fMRI data. The data were collected as a part of the Nathan Kline Rockland Enhanced sample. We decomposed NYC-Q scores using exploratory factor analysis and found that self-reported thoughts clustered into distinct dimensions of content (future related, past related, positive, negative, and social) and form (words, images, and specificity). We used these components to perform an individual difference analysis exploring how differences in the types of self-generated thoughts relate to whole brain measures of intrinsic brain activity (fractional amplitude of low frequency fluctuations, regional homogeneity, and degree centrality). We found patterns of self-generated thoughts related to changes that were distributed across a wide range of cortical areas. For example, individuals who reported greater imagery exhibited greater low frequency fluctuations in a region of perigenual cingulate cortex, a region that is known to participate in the so-called default-mode network. We also found certain forms of thought were associated with other areas, such as primary visual cortex, the insula, and the cerebellum. For example, individuals who reported greater future thought exhibited less homogeneous neural fluctuations in a region of lateral occipital cortex, a result that is consistent with the claim that particular types of self-generated thought depend on processes that are decoupled from sensory processes. These data provide evidence that self-generated thought is a heterogeneous category of experience and that studying its content can be helpful in understanding brain dynamics.

## Introduction

Intrinsically driven neural activity is a fundamental property of the nervous systems of many different species, including humans [1], [2]. The structure of these intrinsic fluctuations in activity can be characterized by imaging techniques such as functional connectivity analysis of resting-state functional magnetic resonance imaging (rs-fMRI) data [3]. Although studies have demonstrated a relationship between mental processes and neural fluctuations by modulating the internal state [4]–[6], the relationship to naturally occurring self-generated thought remains less well defined [7]. Here, we consider the possibility that the temporal dynamics of rs-fMRI data are associated with the patterns of self-generated thought that different individuals experienced at rest. At least in humans we know that individuals vary in their experiences over time (for reviews see [8], [9]) and that self-generated thoughts with no relation to perceptual input are correlated with changes in neural operation [10]–[14]. The current study set out to develop a questionnaire on the content of self-generated thought that could be used to explore the relationship between on-going thought and variations in the rs-fMRI signal.

The association between experiential reports and changes in intrinsic neural activity can be understood in the context of two broad theoretical positions on self-generated thought and its neural correlates. One is that an assembly of regions focused on the medial surface of the posterior and prefrontal regions, commonly known as the ‘default-mode network’ [15], [16], play an important role in self-generated thoughts, as has been indicated by a number of studies showing activity in this system during states such as daydreaming, mind wandering, and other forms of self-generated mental experience [13], [14], [17]. Alternatively, evidence has shown that multiple networks recruited in the service of an external task showed coordinated activity during the resting-state [3], [18]. More generally, recent component process accounts of self-generated experience suggest that it is the result of the interaction of multiple different systems [7].

For example, prior work suggests at least two ways in which neural processes may contribute directly to self-generated experience. Certain neural processes may contribute directly to the occurrence of this experience, by supporting particular content or forms of mental content. For example, integrity in self-generated thought may depend on the capacities to buffer and sustain cognition, which are thought to allow self-generated cognition to form a coherent train of thought [8], [13], [19]–[21]. Alternately, neural systems may contribute indirectly to self-generated thought, for example, because they compete with processes that are more directly implicated in the state. Studies have found that the processing of sensory information is reduced during self-generated thought, a process known as perceptual decoupling [11], [21] suggesting that competition occurs between on-going sensory input and self-generated thought. Reduced neural activity in sensory regions could, therefore, facilitate greater integrity in a self-generated thought [7], [21] (though see [22] for an alternative perspective).

The aim of this study was the development of a retrospective measure of the content of the self-generated thoughts that participants experienced and to explore its relationship to the brain at rest. To this end we created a self-report assessment of the content and form of an individual's self-generated thoughts and incorporated this instrument into the Enhanced Nathan Kline Institute - Rockland Sample (eNKI-RS). The eNKI-RS is a large-scale community-ascertained lifespan neuroimaging study that employs deep phenotyping and data is openly shared as it is being acquired (http://fcon_1000.projects.nitrc.org/indi/enhanced/). Immediately after finishing the scanning session, participants completed the New York Cognition Questionnaire (NYC-Q), a self-report measure of the content and form of the thoughts they experienced during the scanning session. Using exploratory factor analysis (EFA), we identified a number of subscales for the NYC-Q that described the patterns of self-generated that participants experienced at rest. Next we used these scores to guide the results of three whole-brain analyses of the rs-fMRI data: fractional amplitude of low frequency fluctuations (fALFF), regional homogeneity (ReHo), and degree centrality (DC). Each of these complementary analysis methods characterizes a different aspect of brain function, and thus allows us to explore different types of associations between self-generated thoughts and intrinsic neural activity. fALFF quantifies the power of low-frequency blood oxygen-level dependent (BOLD) fluctuations (which drive functional connectivity measures [23]) relative to the power of fluctuations at all other measured frequencies, and has been interpreted as a normalized (for physiological noise) measure of the local, intrinsic low frequency oscillations (LFO) of the BOLD signal (e.g., [24], [25]). ReHo measures the correlation between the activity of a voxel and its neighbours, and can be taken as an index of local functional coherence [26]. DC is a graph-theoretic measure of network structure based on connectivity between voxels, and has been used previously to identify brain regions that act as highly-connected “functional hubs” [27]. For each measure, we identified significant clusters of voxels associated with scores on each NYC-Q subscale. Following [28] we then used these clusters as seeds in a functional connectivity analysis, which allowed us to interpret the membership of each cluster within the context of large-scale functional networks at the group level.

Two broad perspectives guided our theoretical analysis of the link between our self-report measure and the dynamical behaviour of the brain at rest. If the default-mode network is the primary neural contributor to self-generated experience, then correlations between the content and form of experience with fALFF, ReHo, and DC should be restricted to regions within this network. Alternatively, if the behaviour of many different networks contributes directly, or indirectly, to different elements of self-generated thought, then associated changes in intrinsic neural processes should not be constrained to regions within the default-mode network. Although these results may not specify in precise detail the neural processes that contribute to a specific aspect of self-generated thought, they would shed light on the more general question of the widespread nature of neural processes that contribute to self-generated thought, and would also provide preliminary evidence that the dynamical behaviour of the brain at rest can *in principle* be illuminated through an examination of the thoughts that occur during the same period.

## Methods

### Participants

166 participants (102 females; ages 18–60, 1st quartile: 23.25, median: 39, 3rd quartile: 50.75) were recruited as part of the NKI Enhanced Rockland Sample (eNKI-RS)[29]. Although the eNKI-RS includes younger and older participants, only those between 18 and 60 years old were considered for our subsample. This criterion limits the variance introduced by very young and very old participants. As a community representative sample, the eNKI-RS has minimal exclusion criteria, and thus includes participants meeting criteria for one or more DSM-IV-TR Axis 1 diagnoses. Within our subsample, 85 participants (51.2%) met criteria for at least one such diagnosis at some time during their life, which is comparable to the lifetime risk reported by the largest US survey to date (50.8% at age of 75 years – see [30]). Complete information about psychiatric diagnoses in our sample can be found in the File S2. The inclusion in our analysis of data from participants with psychiatric diagnoses allowed us to sample from people with a wide range of mental experiences, though examination of how these experiences may differ due to diagnostic status is beyond the scope of this study. Out of the initial sample, MRI data for only 124 participants were available at the time of the analysis. Three of those had missing or corrupted data, resulting in a final MRI sample of 121 datasets (80 females; age: 18–60, 1st quartile: 23, median: 40, 3rd quartile: 49).

### Ethics Statement

Institutional Review Board Approval was obtained for this project at the Nathan Kline Institute (Phase I #226781 and Phase II #239708) and at Montclair State University (Phase I #000983A and Phase II #000983B). Written informed consent was obtained for all study participants.

### The New York Cognition Questionnaire (NYC-Q)

**Figure 1:**
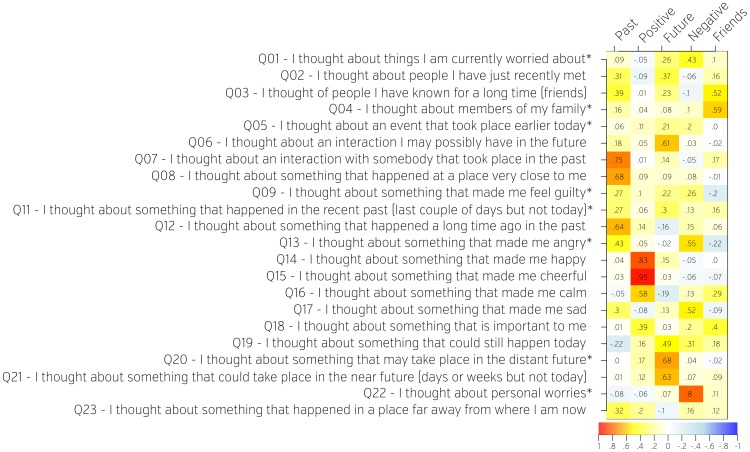
Factor loadings on the questions from the first section of the NYC-Q describing the content of self-generated thoughts. Questions (rows) were decomposed into factors (columns) using Exploratory Factor analysis. Factors were named based on subjective interpretation of the loadings. Weights (how much each question contributes to each factor) are represented both numerically as well as on a colour scale. Questions adapted from DSSQ are marked with an asterisk.

The *New* York Cognition Questionnaire (NYC-Q) is a self-report tool that assesses thoughts and feelings experienced while performing a particular activity. It consists of two sections, the first containing questions about the content of thoughts, the second containing questions about the form that these thoughts take. The NYC-Q is an elaboration of a prior measure known as the thinking component of the Dundee Stress State Questionnaire (DSSQ; see [31]), a measure which we have successfully used to discriminate neural processes associated with self-generated thought in terms of both electroencephalographic measurement [12] and pupillometry [32]. Our primary aim was to extend this measure to encompass elements of self-generated thought that have been studied in the literature but that are not addressed in the DSSQ. For example, individuals differ in the content of self-generated thoughts, including their tendency to engage in mental time travel to the future and past [33]–[35], whether their thoughts are positive or negative [36], and how their thoughts relate to themselves and others [37]. The DSSQ already contains several items that address these content differences (marked by an asterisk in Figure 1), and we developed additional items to provide a more comprehensive account of each content type. Questions from the original DSSQ and these new content questions were grouped and presented as the first section of the NYC-Q. For each question subjects were asked to indicate how well each statement described their thoughts on a scale from 1 (“Completely did not describe my thoughts”) to 9 (“Completely did describe my thoughts”).

In addition to differences in content, studies have shown that participants can differ in the form of self-generated thoughts they engage in: whether they are based on imagery or words [38] and whether they are orientated towards a specific goal [37]. To examine differences in the form of thought, we developed eight questions that are focused on three dimensions: imagery, words, and goal-directed. These questions were presented as the second section of the NYC-Q. The questionnaire used in the current study is available in the Supplementary Materials (Protocol A in File S1), and online at https://github.com/NeuroanatomyAndConnectivity/NYC-Q.

All 166 subjects completed the questionnaire at the end of MRI scanning session (see below for protocol details). One of the questions, “Q10 - I thought about an event that may take place later today”, was included in the questionnaire accidentally and was removed from the analysis due to its similarity to question “Q19 - I thought about something that could still happen today”. The remaining questions (from all 166 subjects) were decomposed using Exploratory Factor Analysis (EFA) to find interpretable latent components. This analysis was applied to each of the two sections of the NYC-Q separately. The number of factors to calculate was estimated using Parallel Analysis [39] a data-driven, permutation based approach that does not require subjective judgement by the researcher. Factors were estimated using principal axis factor analysis. The question answers were not normally distributed (see Figures A and B in File S1), but principal axis estimator is particularly robust to non normal input variables [40]. We also used the oblimin rotation [41], which allow for oblique (i.e., non-orthogonal) factors. Individual-level scores were calculated using the method developed by ten Berge et al., [42]. This approach belongs to a family of exact (also known as refined) score methods (as oppose to coarse score methods) and works by obtaining a weight matrix W transforming individual-level question scores into individual-level factor scores. There are multiple alternative ways to define such matrix - the main difference between the two classes of methods is that the coarse score methods use selected salient items from the item coefficient matrix to perform the transformation instead of all items. In addition to using all items the ten Berge method estimates the matrix W in such way that correlations between individual-level scores are constrained by the correlations between the factors. Since we used the *oblimin* rotation which allows latent variables can be oblique, the method to calculate individual-level factor scores had to be able to preserve correlations between factors. To encourage future collaborative improvement of NYC-Q as well as to promote transparency of our methods, the raw subject scores, the R code used to perform EFA, and the questionnaire itself are available online at https://github.com/NeuroanatomyAndConnectivity/NYC-Q.

### MRI data acquisition

All data (including the MRI scans) were collected as part of the eNKI-RS data sharing initiative [29]. The following scans were acquired for each participant (in a single 1 h long session):

1. MPRAGE (TR = 1900 ms; voxel size = 1 mm isotropic)
2. Resting-state fMRI (TR = 645 ms; voxel size = 3 mm isotropic; duration = 10 minutes)
3. Resting-state fMRI (TR = 1400 ms; voxel size = 2 mm isotropic; duration = 10 min)
4. Resting-state fMRI (TR = 2500 ms; voxel size = 3 mm isotropic; duration = 5 min)
5. Diffusion Tensor Imaging (137 direction; voxel size = 2 mm isotropic, duration = 6 min)
6. Visual Checkerboard Stimulation (TR = 645 ms; voxel size = 3 mm isotropic; duration = 2.5 min)
7. Visual Checkerboard Stimulation (TR = 1400 ms; voxel size = 2 mm isotropic; duration = 2.5 min)
8. Breath Holding (TR = 1400 ms; voxel size = 2 mm isotropic; duration = 10 min)

During the resting-state fMRI (rs-fMRI) scan, participants were instructed to: “Keep your eyes open and stay awake” before each resting-state scan. Eyes open was chosen over eyes closed to prevent subjects from falling asleep. During the scan a fixation cross, white on black, approximately 4 degrees visual angle was presented, to discourage eye movement.

A computerized version of the NYC-Q was administered at the end of the whole MRI scanning session. For MRI analysis we used the MPRAGE scan and the rs-fMRI scan with a TR = 2500 ms. This data was chosen due to the procedural proximity (∼21 minutes instead of 26 or 36 minutes) of its acquisition to the administration of the questionnaire. The subjects were instructed to consider thoughts from the whole hour-long scanning session when filing in the questionnaire.

### MR data processing

#### Preprocessing

Cortical surface reconstruction was performed on the T1-weighted scans using FreeSurfer 5.1 [43]–[52] followed by iterative manual inspection and correction of the cortical segmentations.

The first four volumes of each EPI sequence were removed to insure that the analysed data had reached T1 equilibrium. This was followed by slice-timing and motion correction using AFNI [53]. X, Y, Z displacement as well as the three axis rotations were used to calculate the mean frame displacement (FD), characterizing movement of each participant during the scanning session [54]. These estimates were later used at the group-level to account for subject specific head motion. An affine transformation from mean EPI image to T1 volume was calculated using BBRegister [55]. Whole brain, cerebrospinal fluid (CSF) and white matter (WM) masks were extracted from the FreeSurfer parcellation and transformed into EPI space (thresholded at 0.5 after interpolation). Principal components of physiological noise were estimated using the CompCor strategy [56]: time series from WM and CSF masks as well as voxels of highest variance were used to extract two sets of principal components (also known as *aCompCor* and *tCompCor*). Outliers in the EPI sequence were discovered based on intensity and motion parameters (ArtDetect - http://www.nitrc.org/projects/artifact_detect/). This was followed by denoising of the time series using a general linear model (GLM) with motion parameters, CompCor components, and outliers as regressors (note that global signal was not removed). Residual time series were spatially smoothed with 5 mm full width half maximum (FWHM) isotropic kernel (using FSL). Finally the time series were bandpass filtered (0.1 Hz <f <0.01 Hz) using FSL (however, for the fALFF analysis the full spectrum data has been used).

A nonlinear transformation from T1 to MNI template (created from 152 subjects, 1 mm, provided with FSL) was calculated for individual T1 images using ANTs [57]. This transformation was combined with the EPI to T1 transformation to warp the EPI volumes to standard MNI space (with resampling to 2 mm to save storage space and processing time). This process was applied to both band-passed and full spectrum images (for the normalization factor used when calculating fALFF, see below). Additionally, band-passed images were resampled to 3 mm to reduce the memory requirements of DC calculations.

#### First level analysis

For each individual, three derivatives were calculated: fractional amplitude of low frequency fluctuations (fALFF), regional homogeneity (ReHo), and degree centrality (DC). fALFF is the ratio of the amplitude of fluctuations within a frequency band (0.01–0.1 Hz in our case) to the amplitude of fluctuations within the full spectrum of frequencies [25]. The full spectrum was naturally limited by the sampling frequency (TR) and the scan length resulting in the range of 0.003(3) –0.2 Hz. ReHo measures how similar the time series of each voxel is to their immediate neighbours, indicating local synchrony [26]. DC quantifies how many connections each voxel has to all other voxels (a connection is defined as correlation value higher than 95% of all pairwise non-self correlation values) [27]. All individual derivative maps were *z*-scored to allow for group analysis.

#### Group analysis

Voxel-wise univariate GLM analyses were performed using the derivative scores (fALFF, DC, and ReHo) as dependent variables and fit using separate models for each derivative. Independent variables included factor scores from both sections of the questionnaire as well as age, mean FD [54], [58], and sex. The following contrasts were tested: test for positive and negative correlation of each derivative with each of the factors separately, as well as past vs. future factors and negative vs. positive factors (see Results for names and interpretations of all identified factors). To ensure that individual-level data existed for each voxel included on the group-level, the analysis was spatially restricted to the union each individual's whole-brain masks (estimated from EPI using AFNI). Since interpretation of BOLD outside of grey matter is problematic we also applied a grey matter mask (derived from a probabilistic atlas bundled with FSL).

We also performed two additional analyses: one without the motion covariate on the group level, and one where factors from the two sections of the questionnaire were fit using two separate models (therefore not accounting for their shared variance). We present those results in Figures C and D in File S1.

Inference was performed by deriving suprathreshold clusters (cluster forming threshold of *z* > 2.3) and calculating the probability of the size of each cluster given the estimated smoothness of the image, cluster forming threshold, and search volume within the Gaussian Random Field [59] parametric framework. The threshold used to decide which clusters are significant was set to p<0.05 and corrected for the two-tailed nature of the contrasts and the number of derivatives (fALFF, ReHo, DC) resulting in p_α_  = 0.05/2/3 = 0.0083(3). Considering that fALFF, ReHo, DC were all derived from the same timeseries data, questions arise as to whether they are fully independent measures. As such, Bonferroni correction may be too strict, and could lead to excessive Type II errors. Therefore, we also considered a more lenient cluster size threshold of p_α_ = 0.025, a value that does not account for the number of derivatives we included, although does control for the number of voxels tested. Clusters revealed using the exploratory threshold are indicated and should be carefully interpreted in the context of prior results because of their potential to result from Type I error.

#### Post hoc seed analysis

To investigate how the regions identified by our analysis relate to other areas of the brain, for each participant we extracted the average time series from each significant fALFF, ReHo, and DC cluster (obtained either using the multiple comparison corrected or exploratory threshold) and calculated whole-brain correlation maps for each seed (seed-based analysis similar to one performed in [28]). Correlation maps for each subject were then Fisher's *r*-to-*z* transformed and used in a voxel-wise one-sample *t*-test to calculate group-level patterns of connectivity.

All software workflows were defined in Nipype [60], preprocessing was done using Brain Imaging Pipelines (https://github.com/INCF/BrainImagingPipelines); fALFF, ReHo, and group-level analyses were calculated using pipelines from the C-PAC (http://fcp-indi.github.io/) software package. All code is available on https://github.com/NeuroanatomyAndConnectivity/NYC-Q.

## Results

### Latent Factors in the NYC-Q

The factor decomposition analysis revealed a total number of eight factors describing the content and form of self-generated thoughts. Parallel analysis indicated that the first section of the questionnaire would be best described by five factors, and the second section by three factors. Factor models for both sections revealed good fits: chi squared (131, n = 166)  = 234.17, *p*<6.3e-10 and Root Mean Square Error of Approximation (RMSEA)  = 0.075 (95% CI 0.055–0.083) for the first section, and chi squared (7, n = 166)  = 20.11 *p*<0.0053 and RMSEA = 0.109 (95% CI 0.054–0.162) for the second section. Factors for the first section explained 53% of the variance, and factors for the second section explained 52% of the variance.

**Figure 2:**
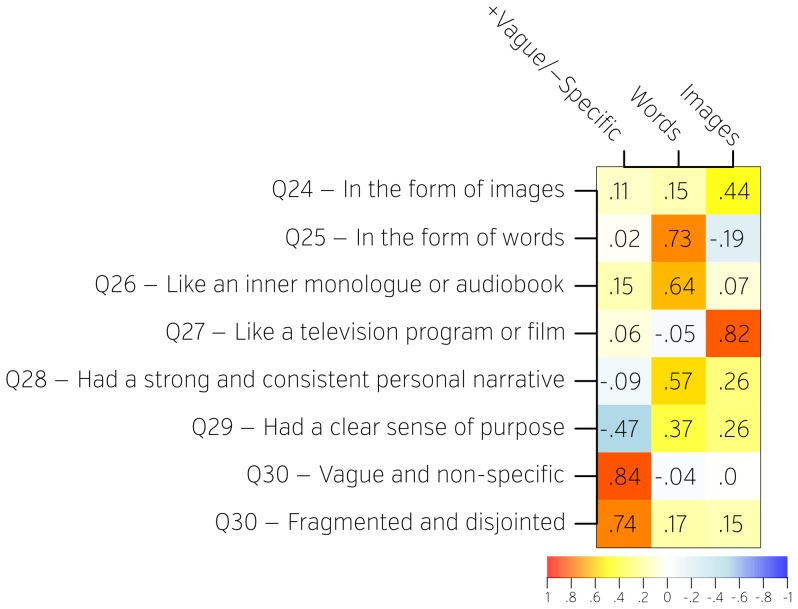
Factor loadings on the questions recovered from the second section of the NYC-Q describing the form of self-generated thoughts. Questions (rows) were decomposed into factors (columns) using Exploratory Factor analysis. Factors were named based on subjective interpretation of the loadings. Weights (how much each question contributes to each factor) are represented both numerically as well as on a colour scale.

By examining questions that load highly on a given factor, we were able to name and interpret each factor. From the first section, we identified factors for thoughts about the past (Past), thoughts about the future (Future), positive thoughts (Positive), negative thoughts (Negative), and thoughts about close social relationships (Social Cognition). From the second section, we identified factors for thoughts in the form of words (Words), thoughts in the form of images (Images), and the specificity of thought (+Vague/-Specific). Factor loadings are presented in Figures 1 and 2. Distributions of each factor loadings across subjects are presented in File S2.

**Figure 3:**
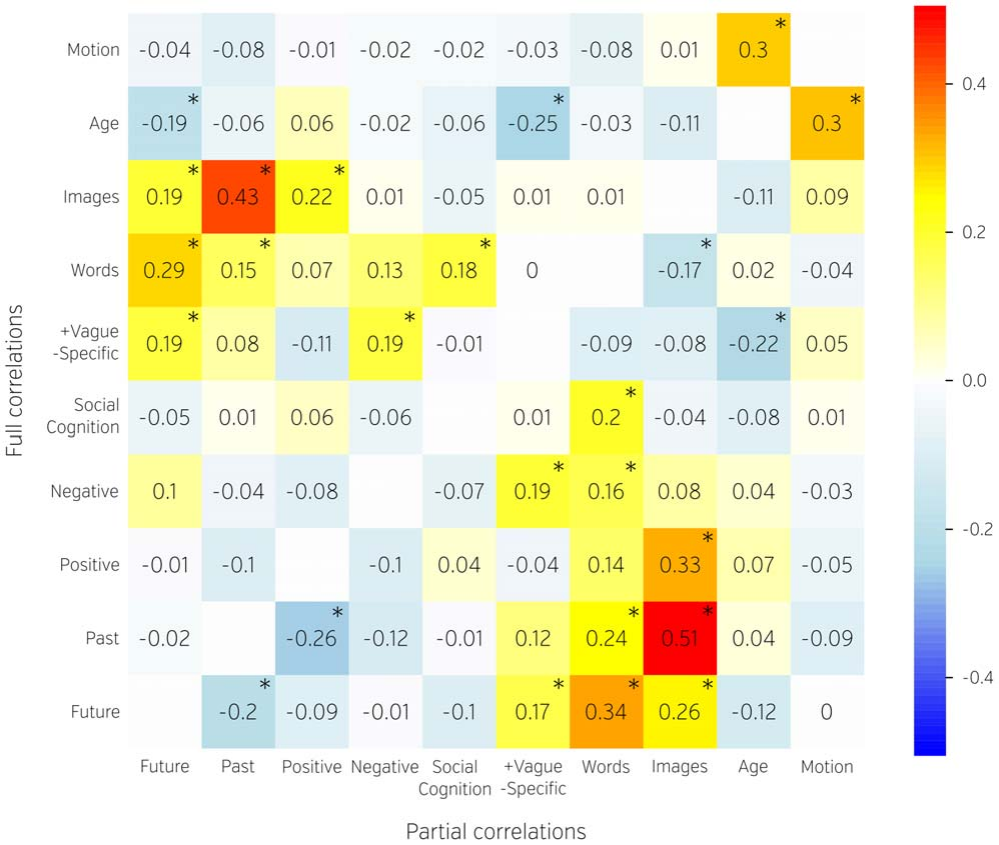
Partial (lower diagonal) and full (upper diagonal) Pearson correlations between age, head motion and estimated factors of the NYC-Q. Numbers and colours represent r values. Significant correlations (two-tailed p = 0.025) are marked with an asterisk.

Partial and full Pearson correlation analysis of age, head motion (mean FD) and factors revealed a number of noteworthy relationships (see Figure 3). Older participants reported greater specificity in their thoughts. Thinking about the past correlated positively with thinking in words as well as images, but negatively with thinking about the future and positive thinking. This is consistent with prior studies indicating that unhappy mood is linked to past-related thoughts [35], [36]. Thinking in words and images correlated with most of the five factors derived from the first section of the questionnaire, although thinking in images was not significantly related to negative or social thought. Participants whose thoughts were also less specific and more vague reported negative thoughts. Head motion was not correlated with any of the derived thought domains.

It is also worth noting that positive and negative thinking was not perfectly anticorrelated. Neither was thinking about the future and thinking about the past. This seemingly counterintuitive fact can be explained by the nature of the measurement. The questionnaire is asking about thoughts across an hour-long session and therefore it is not unlikely that participants could think both about future and past as well positive and negative events. If we were using thought probes to look at individual thoughts it would have been more likely that a single thought would be reported either positive or negative, but not both at the same time. Additionally subjects could have been thinking about the present or neutral events which would yield similar result.

### Neuroimaging Findings

**Figure 4:**
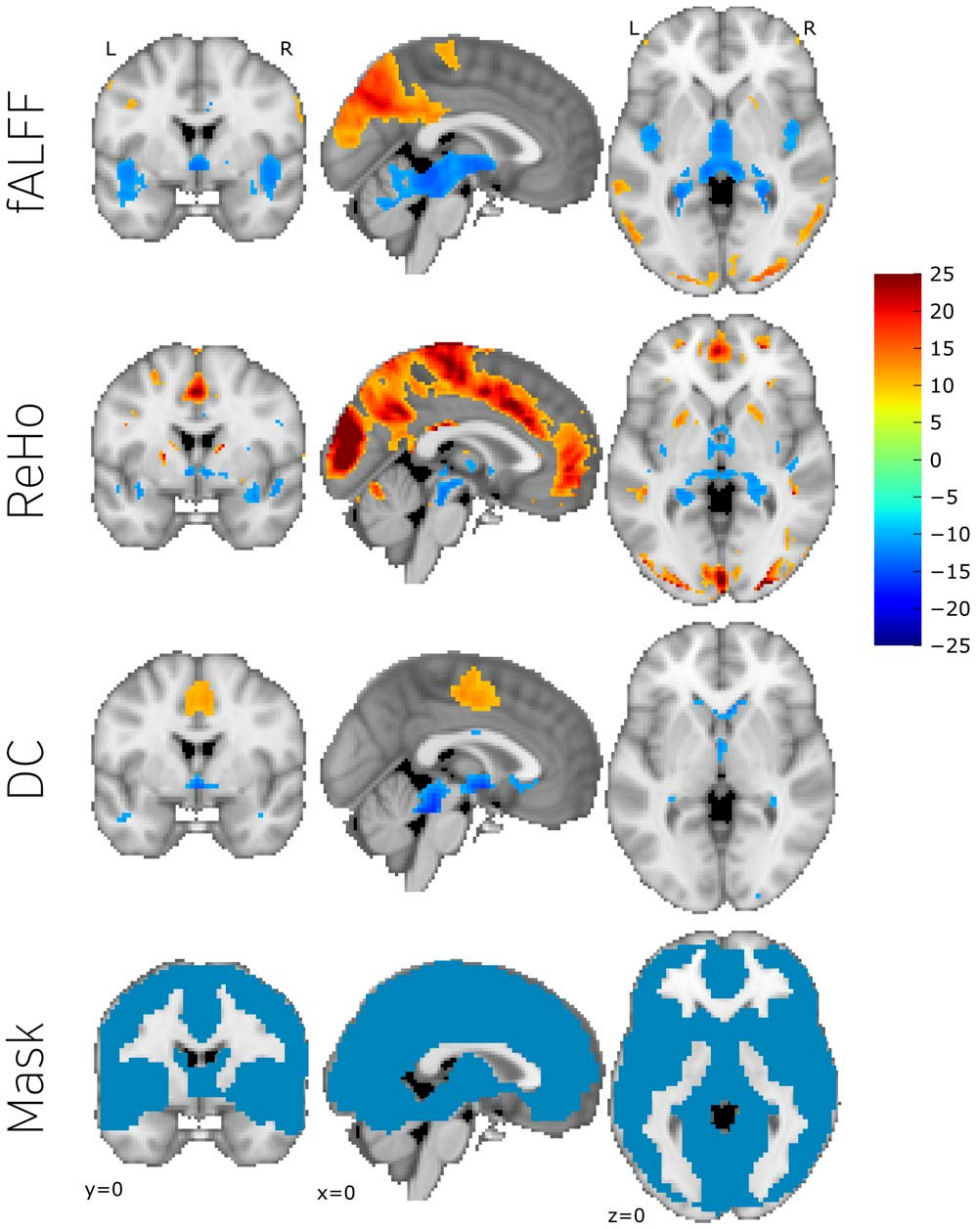
Spatial distribution of fALFF, ReHo, and DC measures across subjects. Each map was obtained from a one sample t test, converted to z values and thresholded ad Z = 10 (for visualisation purposes). The bottom row features the data inferred mask used in the group analysis. pCC and mPFC show high ReHo values and mPFC show high fALFF values. Those are the major hubs of DMN which suggests that even without relating the measures to the questionnaire results DMN plays an important role in brain activation at rest.

**Figure 5:**
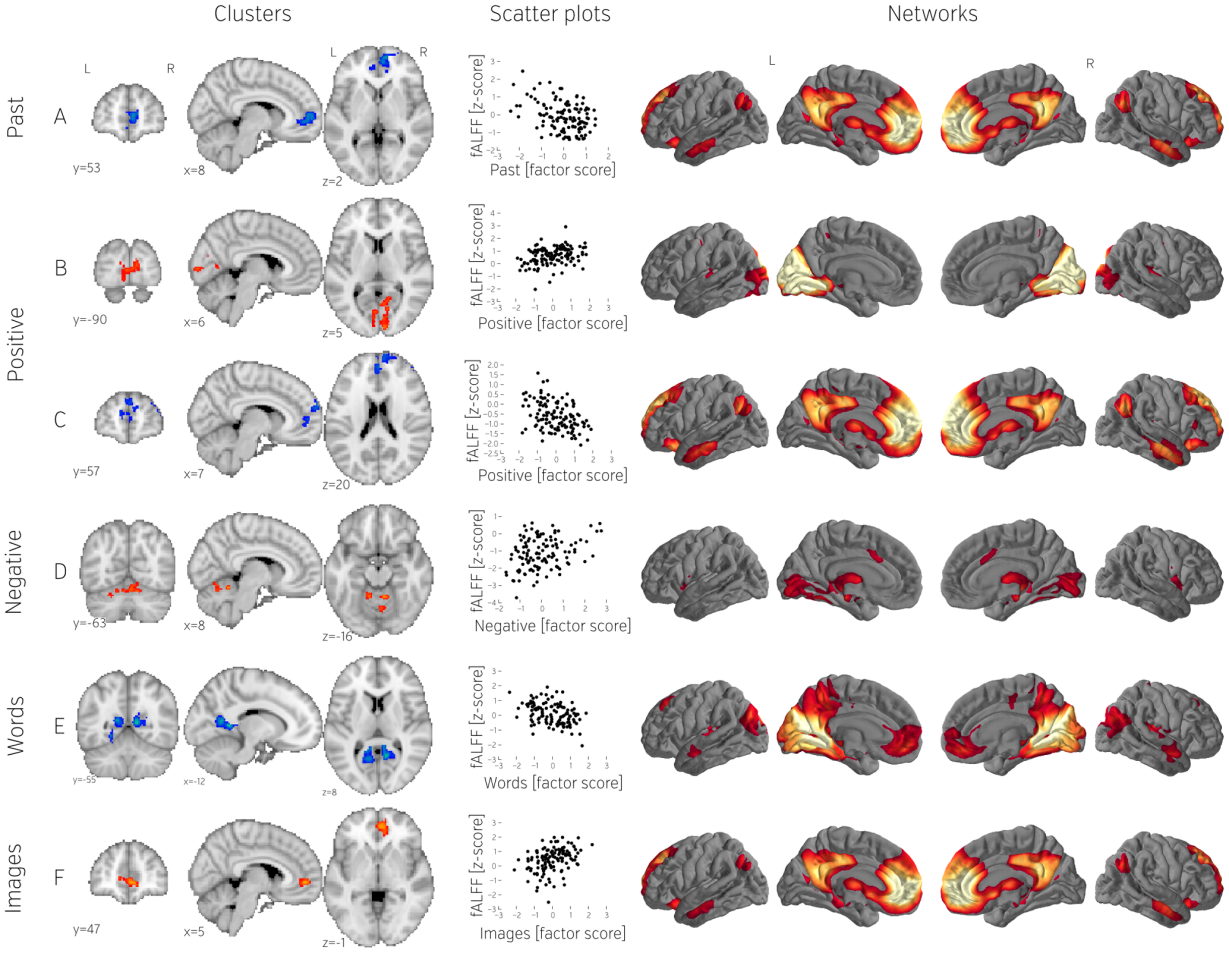
Significant fALFF clusters (A–F), scatterplots showing relation between dependent variables (mean fALFF values) and contrast scores (questionnaire factors), and networks obtained by seeding with the corresponding cluster. All derivatives have been z scored. All scatter plots represent the whole population (n = 121). Note that only clusters that passed the conservative multiple comparison corrected threshold are shown in this figure.

**Figure 6:**
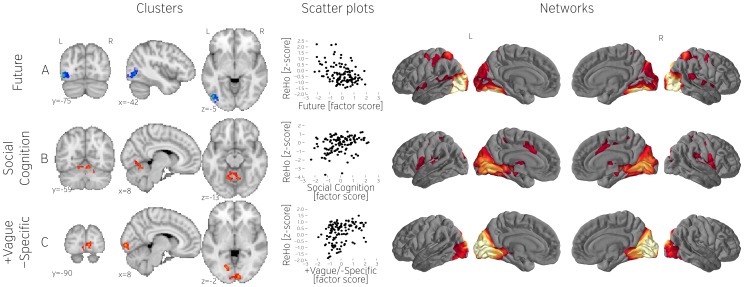
Significant ReHo clusters (A–C), scatterplots showing relation between dependent variables (mean ReHo values) and contrast scores (questionnaire factors), and networks obtained by seeding with the corresponding cluster. All derivatives have been z scored. All scatterplots represents the whole population (n = 121). Note that only clusters that passed the conservative multiple comparison corrected threshold are shown in this figure.

We begin the presentation of the neuroimaging results by looking at patterns of resting-state measures that are consistent across the population. This analysis was performed without taking into consideration their relation to NYC-Q factors. One-sample *t*-tests of fALFF, ReHo, and DC maps revealed higher ReHo values in posterior cingulate cortex (pCC) and medial prefrontal cortex (mPFC) and higher fALFF values in pCC (Figure 4), these regions form what is known as the core DMN. This group analysis, therefore, shows that these elements of the DMN play a role in brain activation at rest, which is consistent with the literature [15]. Analysis of the relation of rs-fMRI data the identified questionnaire factors revealed a number of regions (Figures 5 and 6) and is discussed in detail below. All contrasts are available in the NeuroVault database http://neurovault.org/collections/16/. Coordinates and statistical parameters of all significant clusters are described in Tables 1 and 2.

**Table 1:** Location and statistical parameters of clusters obtained using the multiple comparison corrected threshold.

Cluster	Derivative	Size [vox]	P(size|H0)	Peak [z]	Max [mm]			Centre of Gravity [mm]		
					X	Y	Z	X	Y	Z
5A	fALFF	702	0.0000868	4.44	10	46	−4	8.59	52.8	2.16
5B	fALFF	636	0.000805	3.67	8	−94	4	4.62	−78.3	4.78
5C	fALFF	1185	0.0000025	4.45	34	46	38	13.3	52.6	18.2
5D	fALFF	347	0.00426	3.68	8	−50	−16	−2.76	−61.6	−19.2
5E(right)	fALFF	344	0.00662	4.59	12	−54	10	11.8	−52.6	9.86
5E(left)	fALFF	552	0.000198	4.62	−14	−48	6	−14	−57.4	4.76
5F	fALFF	345	0.00781	4.18	4	50	0	2.91	47.1	0.847
6A	ReHo	287	0.00348	4.58	−46	−74	−6	−41.3	−75.8	−5.07
6B	ReHo	282	0.00513	3.4	−8	−62	−12	2.25	−58.6	−14.9
6C	ReHo	350	0.00176	4.22	−16	−72	−4	−3.3	−84.7	−3.16

Cluster labels correspond to Figures 5 and 6.

**Table 2:** Location and statistical parameters of additional clusters obtained using the liberal threshold the more liberal threshold not corrected for the number of derivatives.

Cluster	Derivative	Size [vox]	P(size|H0)	Peak [z]	Max [mm]			Centre of Gravity [mm]		
					X	Y	Z	X	Y	Z
7A	fALFF	283	0.0199	3.79	−24	−60	−10	−26.2	(61.5	(8.18
7B	fALFF	290	0.0189	4.05	44	(34	12	50.7	(37.1	14.9
7C	fALFF	326	0.00958	4.98	46	(8	(4	45.1	(6.13	(0.726
7D	fALFF	294	0.0112	3.93	40	−66	50	45.2	−61.6	43.6
8A	ReHo	217	0.0187	3.87	−12	−36	70	−11.4	−34.5	69.4
8B	ReHo	234	0.0119	4.5	−12	−38	72	−12.2	−34.6	71.6
9A	DC	240	0.0241	3.88	21	−78	−27	23.7	−70.4	−26.1

**Figure 7:**
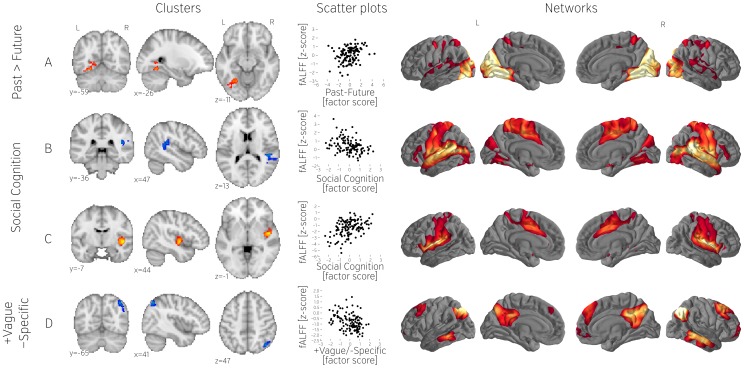
Additional fALFF clusters found using the more liberal threshold not corrected for the number of derivatives. From left to right: location of the clusters (A–D), scatterplots showing relation between dependent variables (mean fALFF values) and contrast scores (questionnaire factors), and networks obtained by seeding with the corresponding cluster. All derivatives have been z scored. All scatterplots represents the whole population (n = 121).

**Figure 8:**
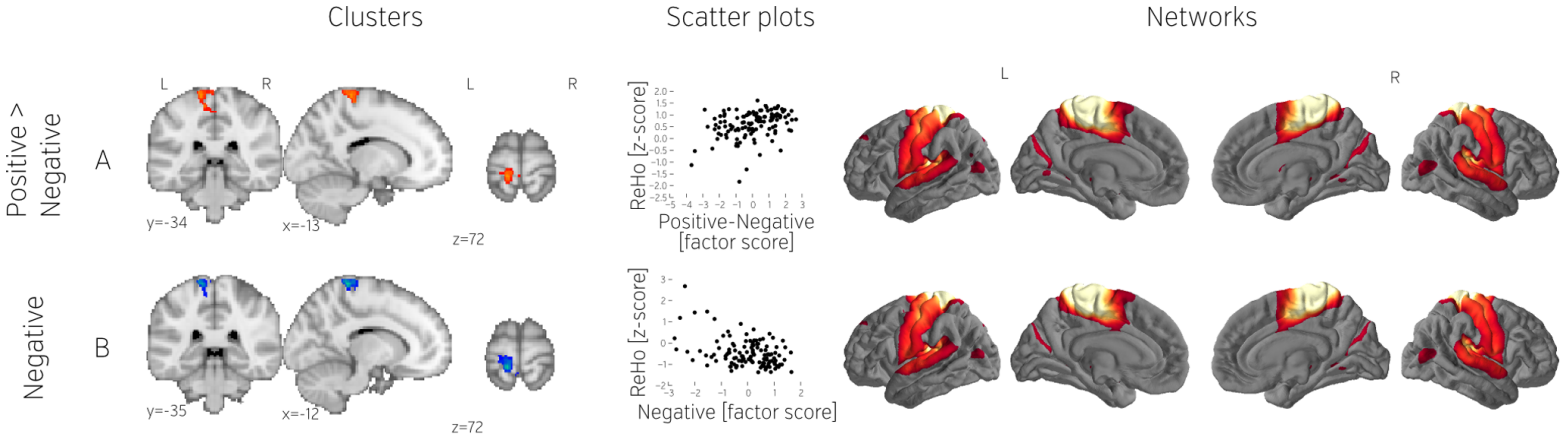
Additional ReHo clusters found using the more liberal threshold not corrected for the number of derivatives. From left to right: location of the clusters (A–B), scatterplots showing relation between dependent variables (mean ReHo values) and contrast scores (questionnaire factors), and networks obtained by seeding with the corresponding cluster. All derivatives have been z scored. All scatterplots represents the whole population (n = 121).

**Figure 9:**
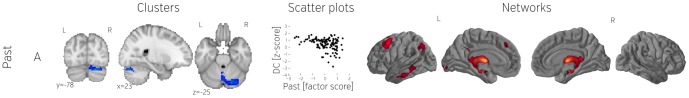
Degree Centrality cluster found using the more liberal threshold not corrected for the number of derivatives. From left to right: cluster location, scatterplot showing relation between dependent variables (mean Degree Centrality values) and contrast scores (questionnaire factors), and the network obtained by seeding with the cluster. All derivatives have been z scored. All scatterplots represents the whole population (n = 121).

Cluster labels correspond to Figures 7, 8 and 9.

#### Fractional Amplitude of Low Frequency Fluctuations

A number of brain regions correlated with either lower or higher degree of low frequency fluctuations. Thoughts about the past were associated with a cluster of voxels in the right mPFC that exhibited lower fALFF (Figure 5A). Functional connectivity from this region presented a pattern with large clusters in both mPFC and pCC — the two key hubs of the default-mode network. We also identified a region in the primary visual cortex that showed a positive relationship between fALFF and positive thoughts (Figure 5, Cluster B). Functional connectivity suggested its involvement in a medial occipital network. We found a cluster in the dorsomedial PFC that showed a negative relationship of fALFF with positivity of thought (Figure 5C). As with past-related thought, functional connectivity with large clusters in the pCC and mPFC was observed. Negative thoughts, by contrast, were associated with greater fALFF in a region of the cerebellum (Figure 5D) which was found to be functionally connected to both the thalamus and a region in the mid cingulate cortex. Two clusters in the left and right caudal posterior cingulate had lower fALFF the more participants characterized their thoughts as being in the form of words (Figure 5E). Functional connectivity from this region was observed with the hippocampal formation and a region of dorsal mPFC. In addition, fALFF in the right paracingulate gyrus was found to positively correlate with thinking in the form of images (Figure 5F). Functional connectivity analysis indicated a default-mode network-like pattern with connections to pCC and the medial temporal lobe.

Using a more lenient threshold, we found a number of additional clusters. This analysis revealed a fALFF cluster in the left temporal occipital fusiform gyrus that was associated with thinking more about past than the future (Figure 7A). We also identified a region of posterior insula in which lower fALFF was associated with social cognition, and subsequent analysis indicated that this was functionally coupled with superior temporal and supramarginal gyrus, as well as regions of somatosensory and motor cortex (Figure 7B). We found a region of mid insula that exhibited greater fALFF with greater social cognition (Figure 7C). Functional connectivity from this region was observed with the insula (bilaterally) and a region of the anterior cingulate, and exhibited relatively less connectivity with somatosensory and motor areas compared to the connectivity of cluster depicted on Figure 7B. Greater specificity (and at the same time lower vagueness) of thought was associated with greater fALFF in a region of the right angular gyrus that was functionally coupled to pCC and lateral regions of parietal and occipital cortex (Figure 7D).

#### Regional Homogeneity

We found evidence linking a change in local synchrony of several brain regions with the content and form of SGTs. Thoughts about the future were associated with lower ReHo in the inferior division of the left lateral occipital cortex (Figure 6A). Functional connectivity analysis suggested that this region participated in a network encompassing bilateral aspects of the visual cortex. We also observed a cluster comprising parts of the cerebellar vermal and paravermal regions I–VI (Figure 6B) that had greater ReHo for individuals who reported high levels of social cognition. This cluster was proximal to the fALFF associated with negative thought, and was also functionally connected to the thalamus and a region in the midcingulate cortex. Additionally we identified a region in primary visual cortex that exhibited higher ReHo in participants who described their thoughts as more vague than specific (Figure 6C). Functional connectivity analysis indicated that this region participated in similar medial occipital network as we observed from cluster shown on Figure 5B.

At the more lenient *p*<0.025 threshold we found associations between negative thoughts and reduced ReHo in the supramarginal gyrus (Figure 8A and B). Seed-based connectivity indicated that this region was functionally coupled to a large region of sensorimotor cortex and a region of the posterior insula.

#### Degree Centrality

Change in terms of whole brain connectivity was correlated only with one factor and only for one brain area. Using the more lenient *p*<0.025 threshold, we found that past related thought was associated with less DC in the cerebellum (Figure 9A). Seed-based connectivity indicated that this area was linked to the thalamus, a region of lateral occipital cortex and a dorsal region of prefrontal cortex.

## Discussion

Our study set out to develop a questionnaire to identify patterns in the content and form of self-generated thought that individuals experience at rest, and to relate these to the dynamic patterns of neural behaviour that occur during an rs-fMRI scanning session. To this end, we created a measure of cognition, the NYC-Q, and decomposed this using exploratory factor analysis. We identified several different categories of self-generated thought related to both their content (social cognition, future, past, positive, and negative) and form (words, images, and specificity). We used these categories of experience to guide three methodologically different whole-brain analyses, which revealed regions whose intrinsic behaviour was differentiable along the dimensions of reported experience. We discuss the nature of the components of self-generated thought determined by our decomposition of the NYC-Q and consider their neural correlates in relation to prior work. Finally, we explore the implications of our results for theoretical perspectives on the brain basis of self-generated thoughts.

### The New York Cognition Questionnaire

Our decomposition of the NYC-Q allows insight into the internal structure of self-generated thought. Importantly, our findings are broadly consistent with the results of recent studies that used experience sampling during a laboratory task. For example, Ruby and colleagues [36], [61] decomposed the contents of multiple experience sampling questions and found that past and future related thought were independent categories of experience. Although the decomposition of the NYC-Q revealed distinct positive and negative elements to thought, Ruby and colleagues [36] found a single dimension of thought anchored at one end by negative experiences, and at the other by positive experiences. Certain differences are inevitable between retrospective and online measures of experience [8], however, both approaches indicate a broadly consistent internal structure to the content of self-generated thought: (i) a clear distinction between past and future temporal foci and (ii) a further distinction between positive and emotional valence. In terms of form, we found dimensions of verbal and visual thoughts that were anti-correlated (*r* = −.17). Inner speech and imagery are thought to be two distinct components of experience [62] and prior work has also found that these dimensions are negatively related during experience sampling studies [63].

We also noted a number of relationships between the dimensions of content and form that are broadly consistent with prior findings. We found that future thought was more strongly correlated with verbal thought (*r* = .34) than was past thought (*r* = .24). Prospection thought was also more positively correlated with words than images (*r* = .24). Together, these results are consistent with Stawarczyk et al., [63] who found future thought was positively related to verbal thought, perhaps because of the role that language plays in planning our future [64]. We also found that past thought was negatively correlated with positive thought, and this is consistent with prior studies indicating that unhappy mood is linked to past-related thoughts [35], [36], [65], [66]. Although research on the internal structure of self-generated thought is still in its infancy, it is encouraging that our decomposition maps well with prior findings in this domain.

### Neural correlates of the content of the New York Cognition Questionnaire

Our study identified the neural correlates of content differences in self-generated thought; in particular their temporal, emotional and social features. Both positive and negative correlations between rs-fMRI derivatives and questionnaire factors were investigated. The interpretation of negative correlations has been problematic in terms of functional connectivity. Even though we also discuss positive and negative correlation, those are not directly related to functional connectivity but the relation between behaviour and fALFF or ReHo measurements. In the context of our study both negative and positive correlations indicate that the neural processes in a particular brain region are informative of (i.e. can predict) the self-reported content of experience. These results could occur for multiple reasons — for example decrease of fALFF (in case of a negative correlation) could arise because of upstream inhibition by other regions. Recently Etkins and colleagues applied trans cranial magnetic stimulation to the dorso-lateral pre-frontal cortex to demonstrate that this region exerts a controlling influence on fALFF activity in the medial pre-frontal cortex [67]. Ultimately, answering the question of what neuronal processes are responsible for less variable BOLD signal (fALFF) and decrease in local synchrony (ReHo) in the context of mind wandering remains open and may require techniques beyond fMRI. Whether the behaviour of a neural regions is positively or negatively related to the content, or form, of experience is important in determining the possible process that underlies any relationship and we consider these in detail in the section on implications. With these limitations in mind we consider our findings in light of prior work on different aspects of self-generated thought.

#### Past/Future

Increasing reports of past-related thought were associated with decreased fALFF in a region of the mPFC. States of mind wandering or daydreaming, are normally characterized by a greater focus on the future than the past, a relationship that has been observed in countries including the United Kingdom, the United States [34], [68], [69], Belgium, Germany [36], [70], and China [71]. It is interesting to note that people who tended to report forms of experience that tend not to be common in today's society exhibited reduced low frequency fluctuations in a core region of the default-mode network (the mPFC).

ReHo in a right lateral region of occipital cortex was associated with less future thought. In an adjacent region fALFF was higher for individuals who reported greater past than future thought. Together, these data suggest that neural activation in left lateral occipital cortex is reduced and less cohesive for individuals who engaged in future related thoughts. Studies suggest that future thought is common under situations when attention need not be paid to visual input [32] and the reduced ReHo in lateral visual cortex, coupled with the reduction in fALFF in an adjacent region could reflect the relative unimportance of immediate sensory input in self-generated thoughts about the future (see section on Implications).

#### Positive/negative emotions

We also found neural correlates for emotional aspects of self-generated thought. Positive experiences showed increased fALFF in a region that participated in a medial occipital network, and decreased fALFF in a region of dorsal mPFC. Several recent meta-analyses have shown that the visual cortex is activated during states of positive affect [72], [73] and this evidence supports our finding that this region is involved in positive mental content at rest. Moreover, the medial occipital cortex plays a direct role in imagery [74], and so it is informative that we found a correlation between positivity and imagery factors of the NYC-Q at the group level. Positive thought was also associated with reduced fALFF in a region of dorsal medial pre-frontal cortex. This region of cortex has been associated with appraisal and expression of negative emotion (for a review see [75]) perhaps because it is important role in conscious threat appraisal [76]. This perspective provides convergent support for our finding that fluctuations in dorsal-medial pre frontal cortex activity are relatively strong for individuals who report an absence of thoughts with positive features (such as calmness).

Negative thoughts, by contrast, were associated with changes in fALFF in regions of the cerebellar vermal and paravermal regions V and VI. This cerebellar clusters had a functional connectivity pattern indicating an association with the thalamus and the anterior cingulate, areas activated together with cerebellar areas during cardiovascular arousal [77], [78] and negative emotional processing [79]. It is important to note that connectivity changes in the lower cerebellar regions VII-X could not have been detected, as those regions were excluded due to only partial coverage in some of the subjects (see group mask in Figure 4), thus obscuring the possible contribution of vermal and lateral cerebellar areas to other emotional functions [79], [80].

#### Social

Social cognition was associated with changes in the fractional amplitude of low frequency fluctuations (fALFF) in the insula: less fALFF was found in a posterior cluster and greater fluctuations were seen in a cluster in the mid-insula. Functional connectivity analysis indicated that both of these regions covaried with a network including the caudal anterior cingulate, regions which together form a network often described as the saliency network [81], [82]. However, relative to the anterior region, the posterior cluster was distinguished by its greater connectivity with regions of the somatosensory and motor cortices.

The insula is an important hub in the brain [83] that allows for the integration of affective and feeling states [84]. Anterior regions of the insula have been hypothesized to be important in social cognition because it allows the affective and somatosensory simulation of the affective states of other individuals, as occurs during states like empathy [85], [86]. Such simulation is not thought to involve the posterior regions of the insula because they receive projections directly from primary somatosensory and motor regions that are important for somato-sensation, but not for simulation related to these states [87]. Our data is, therefore, generally supportive of the role of the anterior insula in social cognition, because individuals reporting such experiences exhibited a shift in the locus of low frequency fluctuations from the posterior towards the anterior regions. Social cognition was also associated with greater ReHo in similar cerebellar vermal and paravermal regions V and VI as it was linked to negative thought. Importantly social and negative thought were uncorrelated at the group level.

### Neural correlates of the form of self-generated thought

We also found neural correlates associated with individual differences in the *form* of self-generated thought, as described below:

#### Images/words

Prior investigations suggest that thoughts in imagery are often the dominant form of mental experience during the resting state [69], [88]. We identified a region in the perigenual cingulate cortex associated with greater thoughts in the form of images. Functional connectivity confirmed this region's participation in the default-mode network, suggesting a role for this network in aspects of self-generated thought related to mental imagery.

A region of the right and left caudal posterior cingulate cortex exhibited lower fALFF for individuals who rated their experiences as related to words. Our functional connectivity analysis suggests that this region has extensive connections to the hippocampus, the pCC as well as the dorsomedial PFC, although it lacked the strong coupling to the ventro medial PFC that is characteristic of the default-mode network. The involvement of the hippocampus in the default-mode network is a matter of debate [89], [90], however, studies suggest that this structure is involved in processes of episodic memory and spatial navigation (for a meta-analysis see [91]), which together are thought to depend on the process of scene construction [92]. Further research will be needed to ascertain why the region of the caudal posterior cingulate cortex exhibits less fluctuation at rest for individuals whose thoughts are verbal in nature.

#### Specific/Vague

Studies have shown that participants tend to report self-generated thoughts that are often specific in nature [69]. Our data found an area of the right angular gyrus that showed greater fALFF for individuals who characterized their thoughts as specific rather than vague. Functional connectivity demonstrated that this region overlapped with the posterior cingulate, yet had a dorsolateral rather than medial focus in the prefrontal cortex that would be characteristic of the default-mode network. This network is similar to the fronto-parietal control network [93]: a neural system that is important in engaging controlled processing across a wide range of different external tasks [94]. Our data could reflect the engagement of control on self-generated thoughts, a process which could facilitate the process of buffering and sustaining that would help support a detailed internal train of thought [7]. This hypothesis is supported by evidence that participants who have greater capacity for control [20], [95], [96] engage in more self-generated thoughts under non-demanding conditions and that these are often goal-focused [37]. By contrast an individuals whose thoughts were vague, rather than specific, exhibited greater neural changes in regions of the medial occipital cortex. This supports the notion that sensory information can compete with internally focused information and so prevent a detailed and specific train of self-generated thought from emerging (see Implications section for further discussion).

### Implications for a neural account of self-generated experiences

Our data found evidence of the importance of the default-mode network during the resting state. At the group level we found that the pCC exhibited both high amplitude fluctuations, as measured by fALFF, and exhibited highly homogeneous neural behaviour, as assessed by ReHo. Likewise, the mPFCs neural behaviour was relatively homogeneous. These data suggest that across our sample the two key hubs of the DMN exhibit behaviour during the resting state that is cohesive. The pCC exhibited high levels of temporal variation in the low frequency range. We also found evidence that the behaviour of regions likely to participate in the default-mode network contained information on the content of an individual's experience at rest. For example, regions in the perigenual cingulate exhibited greater fALFF for individuals reporting high levels of imagery and aspects of the dorsomedial pre-frontal cortex exhibited less activity for individuals whose thoughts did not have a positive tone.

The neural correlates that differentiated individuals in terms of the nature of their self-generated experiences were often in regions that are not members of the canonical default-mode network, such as the visual cortex, the parietal cortex, the cerebellum, and the insula. Some of these regions may directly contribute to the form or content of self-generated experience. Meta-analysis, for example, has shown that the occipital cortex is activated during positive thought [72], [73]. The relation between greater fALFF in the medial occipital cortex and greater positive thought is consistent with this finding (Figure 5B). It is also possible that the association between social cognition and fALFF in the mid-anterior insula reflects a direct role of feelings and affective states in generating thoughts about the self and others (Figure 7C) [85], [86].

A large number of the neural correlates we found reflected negative correlations with different aspects of experience. These types of relationship may reflect competition between many different cortical regions to produce the relatively narrow aspects of conscious experience. For example, an assumption that has grown to prominence in theoretical accounts of self-generated thoughts is that for consciousness to focus inwards in a detailed manner, external input must release its grasp on attentional processes. This phenomenon is known as *perceptual decoupling* [7]. Studies using electroencephalography (EEG) demonstrate that the cortical processing of on-going input is reduced during self-generated thought [10]–[12], in part because of a reduction in the phase locking in the occipital cortex [97]. Based on studies in anesthetized monkeys, it has been shown that the BOLD signal is governed by local field potentials [98]. Based on this mechanism, it is possible that reduced coherent neural activity and lower amplitude activity in the BOLD signal could underlie the reduced cortical processing of sensory information as indexed using EEG that occurs during self-generated thought. In theory, this would help people focus in a detailed manner on self-generated mental events [7], and so could explain the reduction in ReHo medial aspects of occipital cortex for participants whose thoughts were specific rather than vague. It could also account for the relative absence of cohesive neural fluctuations in the medial occipital cortex for individuals whose thoughts were highly specific. Future work that links the electrical information provided by the electro-encephalographic with the BOLD signal provided by fMRI could plausibly illuminate the mechanisms through which intrinsic and extrinsic sources of information interact to produce different forms of conscious experience.

Altogether, our data demonstrate that the relation between the neural changes taking place during the resting state and aspects of self-generated thought is complex. We found that regions could either be correlated or anti-correlated with the content or form of self-generated thought. Moreover, these neural correlates were distributed across many regions of cortex, rather than being limited to only one functional network. Just like many brain networks exhibit intrinsic coordinated activity at rest [3], [18], many may also be associated with the thoughts and feelings that unfold during the resting-state.

### Limitations and future directions

One important caveat of our results is that an association between neural and psychological changes does not provide definitive information on the mechanisms that contribute to any underlying relationship. Our analysis adopted an individual difference approach and so exploited variation across participants as a proximal measure of different forms of experience. Using only a single session of rs-fMRI data, it is impossible to determine whether any association between psychological and neural domains relies on a trait that encourages the expression of that particular type of thought indirectly, or is instead related to the processes that directly reflect the experience itself. Nor does the co-occurrence of change within a psychological domain provide information on what function it plays. There are likely numerous sub processes that underpin a complex psychological state such as having thoughts about the future, governing the specificity of its content, or whether its form was in imagery or words. Furthermore, several rs-fMRI scans were acquired in our protocol, and our analyses only included the final scan, even though the questionnaire asked about thoughts during the whole hour-long scanning session. This selection was made to minimize the time between scanning and retrospective reports, and also to use the scanning sequence that is most common in fMRI studies. It is possible that our analysis would have greater power if participants were presented with the questionnaire directly after the rs-fMRI scan and instructed to limit their reports just to that period of time. This adjustment would increase temporal precision regarding when the experiences occurred and therefore is recommended for future experiments. It is also known that experiences systematically change over the course of an experimental session[99], [100] and so it is possible that certain aspects of our results would be different if we had analysed the first rather than the last resting-state scan. For these reasons, while our decomposition of the NYC-Q demonstrates that there are multiple forms of self-generated thought, and their relation to the resting-state indicates differentiable neural correlates, the precise nature of these mind-brain relationships will require further research. It is likely that examination of the quality of thoughts with different measures, or in different groups or situations, may well yield different patterns of covariance at the neural or subjective-level, and thus a different pattern of results. An appropriate inference to draw from our data is that because we show distinct patterns of experience with reliable neural correlates, self-generated experiences are not a single homogeneous class of mental or brain states, however, the specific neural correlates that we find must be considered as hypotheses that will guide future research, and should not be viewed as definitive accounts of the neural basis of different aspects of the experience.

It is worth noting that in contrast to seed-based functional connectivity [3] and dual-regression methods [101], the analyses we performed did not require a priori assumptions about the specific brain regions or networks involved. Moreover, each of the measures we used (fALFF, ReHo, and DC) provided different, but complementary information about aspects of underlying neural processes, and together they provide an exploratory picture of the complex way in which self-generated experience may map onto intrinsic changes in the brain.

The three measures we used to investigate the temporal behaviour of the BOLD signal at rest exhibited differential sensitivity to our measure of self-generated thoughts. In terms of number and statistical significance, fALFF outperformed ReHo and that in turn did better than DC. It is an open question as to why some measures performed better than others, but one of the characteristics of DC that distinguishes it from other measures is its global nature. fALFF and ReHo are local measures — in the sense that they are using each voxel data (or each voxel's immediate neighbours) independently of each other. DC, on the other hand, takes into account pairwise connections between all voxels. The relatively poor sensitivity of DC in our study might indicate a local rather than global (distributed) nature of the neural correlates of the content and form of self-generated thoughts. It is, however, important to note that the relative sensitivity of the derivative to a particular aspect of cognition depends both on the power of the derivative as well as the nature of the targeted neural process. As we currently do not know how to independently determine the relative spatial scope of the neural processes that are important to self-generated thoughts, it is premature to draw firm conclusions on the relative merits of the techniques we applied in the current investigation.

Finally, we developed the NYC-Q as a tool to measure experience retrospectively. It adds to growing body of similar resources including the Dundee Stress State Questionnaire [31], the Amsterdam Resting State Questionnaire [102] (which has a well developed factor structure and high test-retest reliability), and the Resting State Questionnaire [88]. The retrospective nature of these measures all have the advantage of allowing an individual's thoughts to be estimated without interrupting the stream of consciousness or the collection of physiological data. However, the reliance of all of these measures on memory leads to the concern that biases in recall may reduce their accuracy. In addition to pursuing neural correlates that these tools reveal, we recommend that an important goal for their further development should be their comparison to online techniques that rely less on memory, such as experience sampling (see [8] for a review of these issues). This technique involves interrupting individuals to identify the current contents of thought, and has been used in task-based contexts to illuminate the neural correlates of self-generated thought [13], [14], [103]. It would be useful to identify the overlap between experience sampling indices of the content of thought and the NYC-Q because this would help determine whether the two measures could be used in an interchangeable fashion. Such an experiment would involve interrupting a participant using experience sampling probes and asking questions about the content and form of thoughts immediately preceding the probe [36]. The questions would be a shorter version of the ones used in the NYC-Q. At the end of the rest period the NYC-Q would be administered and results from the thought probes collected during the rest period would be compared with the answers to the questionnaire. As we anticipate that addressing this and related questions in a comprehensive manner would benefit the larger cognitive neuroscience community, we have provided our questionnaire online at https://github.com/NeuroanatomyAndConnectivity/NYC-Q to foster a more integrated and collaborative approach to further investigating this research question.

## Conclusions

Our study found that the content of self-generated thought as measured by the NYC-Q had a consistent structure and that the relationship between these different types of thoughts and neural activity at rest extended across many different regions of cortex. We also found that it was characterised by both positive and negative associations with neural processes. Given the complexity and richness of human experience during the resting-state, it seems plausible that its most likely neural correlate will not to be found in a single neural system. Instead our study suggests that a form of experience with such variety is more likely to arise through the complex interplay of brain regions distributed across the cortex, as well as perhaps multiple large-scale networks. We encourage further exploration of the distinct role of the default-mode network, as well as those of other large-scale networks, in different aspects of self-generated experience.

## Supporting Information

File S1 **Additional figures and the New York Cognition Questionnaire.**
(DOCX)

File S2 **Information about mental illness diagnoses within the sample (Comma Separated Values file).**
(CSV)
