# Supplementary material for: A Correspondence between Individual Differences in the Brain's Intrinsic Functional Architecture and the Content and Form of Self-Generated Thoughts
Source: PLoS One. 2014 May 13;9(5):e97176. doi: 10.1371/journal.pone.0097176 (PMC4019564; doi:10.1371/journal.pone.0097176)
Supplement: File S1 — Additional figures and the New York Cognition Questionnaire. (DOCX) [file pone.0097176.s001.docx]

# S1 Supporting Information File

Protocol A: The New York Cognition Questionnaire

Section 1 [Content]

"We are interested in the thoughts and feelings that you experienced during the MRI. Please indicate which statement describes the thoughts that you experienced while completing the MRI. Indicate the extent to which your thinking corresponded to each item by selecting the relevant option associated with each sentence."

1 (Completely did not describe my thoughts) - 9 (Completely did describe my thoughts)

Q01 - I thought about things I am currently worried about

Q02 - I thought about people I have just recently met

Q03 - I thought of people I have known for a long time (friends)

Q04 - I thought about members of my family

Q05 - I thought about an event that took place earlier today

Q06 - I thought about an interaction I may possibly have in the future

Q07 - I thought about an interaction with somebody that took place in the past

Q08 - I thought about something that happened at a place very close to me

Q09 - I thought about something that made me feel guilty

Q10 - I thought about an event that may take place later today

Q11 - I thought about something that happened in the recent past (last couple of days but not today)

Q12 - I thought about something that happened a long time ago in the past

Q13 - I thought about something that made me angry

Q14 - I thought about something that made me happy

Q15 - I thought about something that made me cheerful

Q16 - I thought about something that made me calm

Q17 - I thought about something that made me sad

Q18 - I thought about something that is important to me

Q19 - I thought about something that could still happen today

Q20 - I thought about something that may take place in the distant future

Q21 - I thought about something that could take place in the near future (days or weeks but not today)

Q22 - I thought about personal worries

Q23 - I thought about something that happened in a place far away from where I am now

Section 2 [Form]

"We are interested in the thoughts you had during the MRI. Please indicate the extent to which each of these statements correctly characterizes your thinking. Please indicate, using numbers, how much the statement characterizes your thoughts during the MRI."

1 (Completely does not characterize my experience) - 9 (Completely does characterize my experience)

During the MRI my thoughts were:

Q24 - In the form of images:

Q25 - In the form of words:

Q26 - Like an inner monologue or audiobook:

Q27 - Like a television program or film:

Q28 - Had a strong and consistent personal narrative:

Q29 - Had a clear sense of purpose:

Q30 - Vague and non-specific:

Q31 - Fragmented and disjointed:


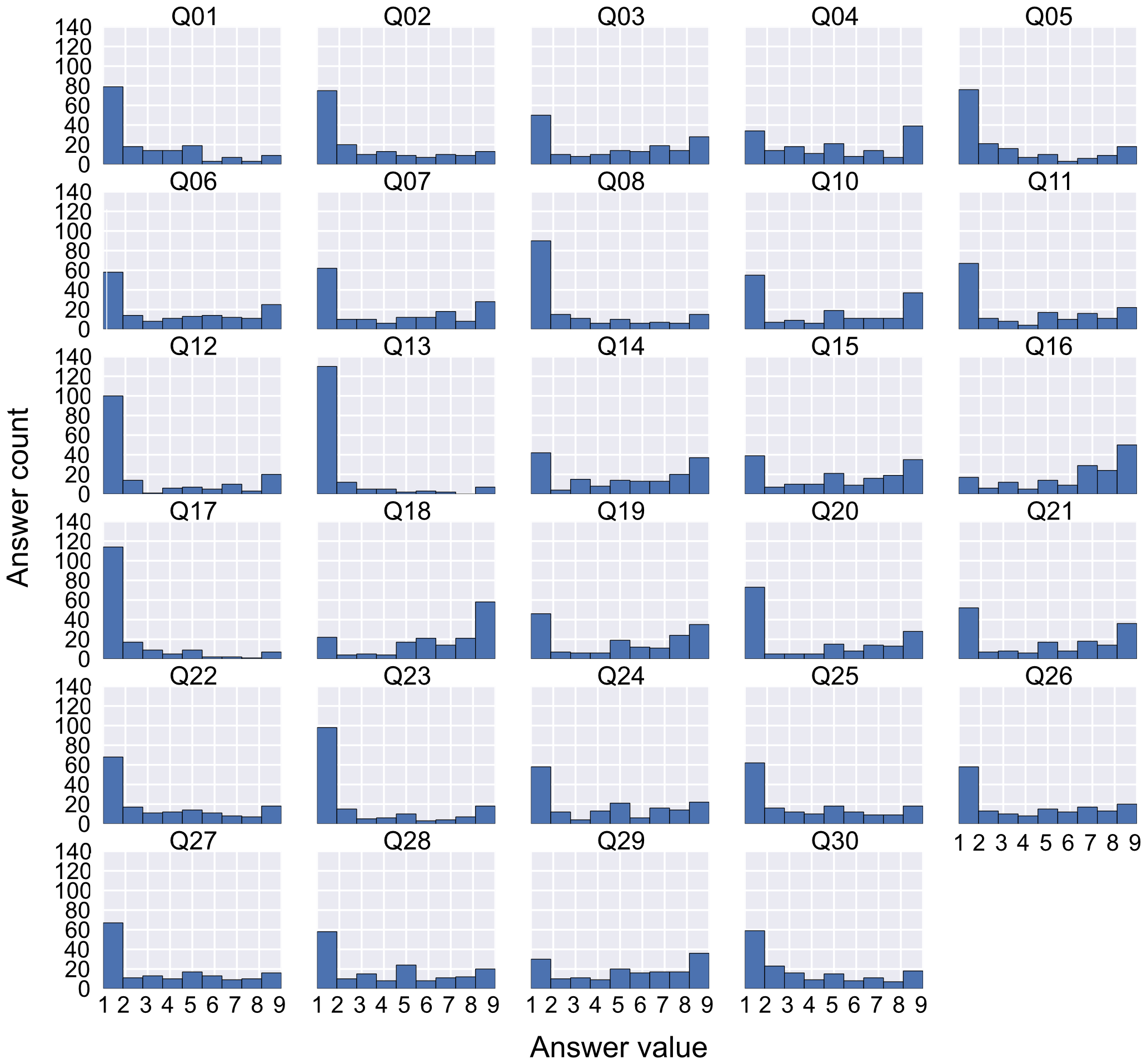


Figure A. Distribution of questionnaire answers.


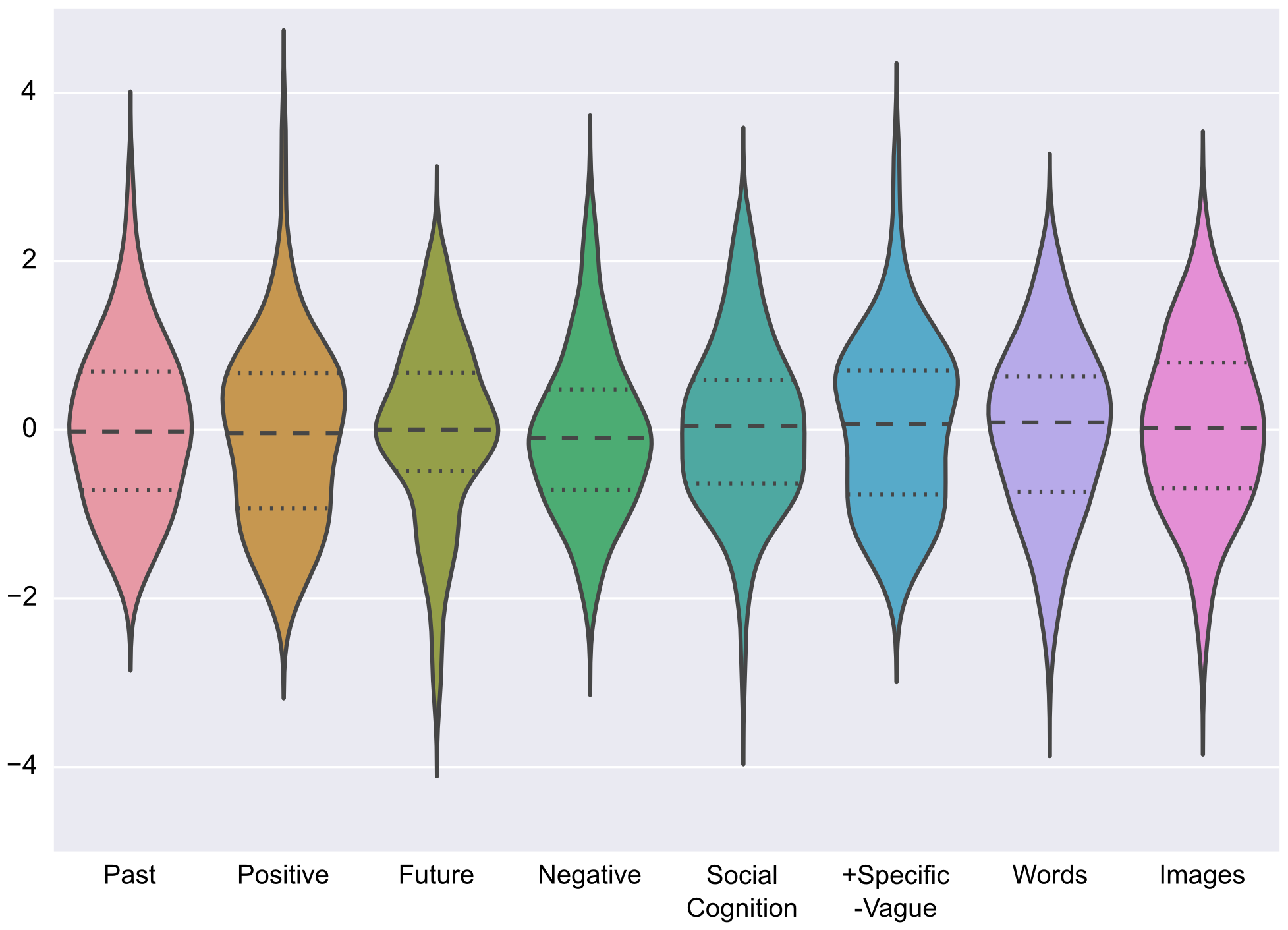


Figure B. Distribution of factor scores.


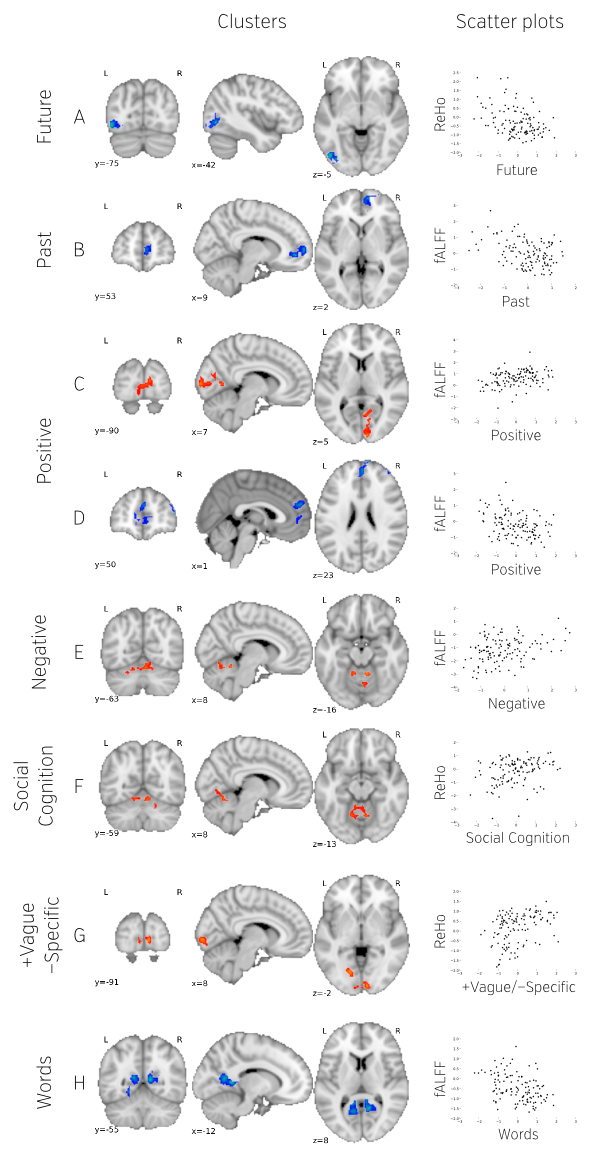


Figure C. Cluster locations, size, and shape after removing mean FD as a confound regressor.


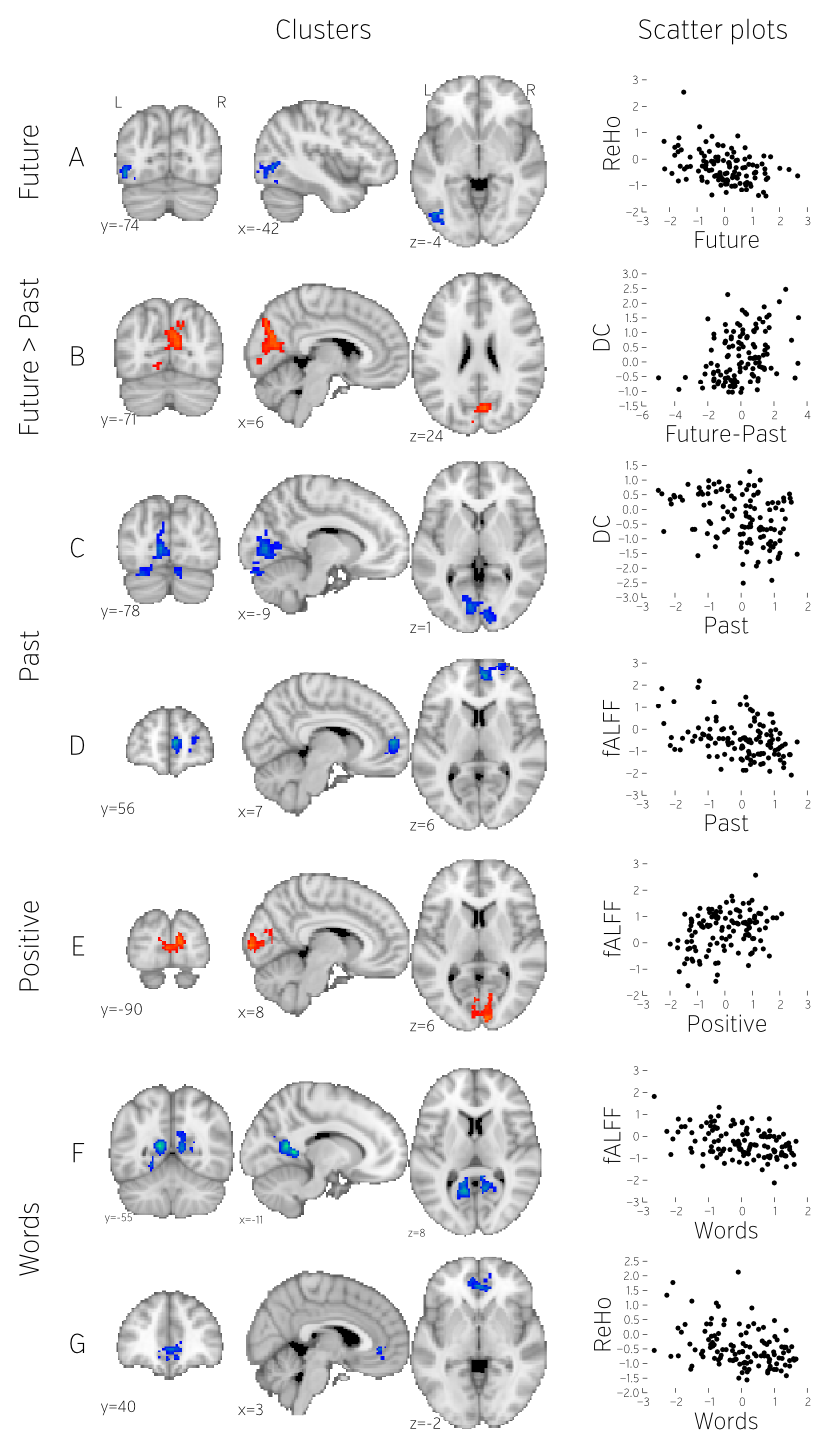


Figure D. Cluster locations, size and, shape after analyzing factors from the two parts of the questionnaire in separate GLMs.
